# School Lunches in Poland—Planned Wastage

**DOI:** 10.3390/foods15173090

**Published:** 2026-08-31

**Authors:** Jan den Boer, Karolina Sobieraj, Emilia den Boer, Gudrun Obersteiner

**Affiliations:** 1 Department of Applied Bioeconomy, Wrocław University of Environmental and Life Sciences, 50-375 Wroclaw, Poland; jan.denboer@upwr.edu.pl (J.d.B.); karolina.sobieraj@upwr.edu.pl (K.S.); 2 Faculty of Environmental Engineering, Wroclaw University of Technology, 50-370 Wroclaw, Poland; emilia.denboer@pwr.edu.pl; 3 Institute of Waste Management and Circularity, University of Natural Resources and Life Sciences, 1190 Vienna, Austria

**Keywords:** food waste, school catering, plate waste, unserved food, school canteens, institutional food services

## Abstract

Food is lost and wasted at different points in the food supply chain, causing serious environmental, social and economic implications. School lunches are a significant source of food waste at the consumption stage. This study, undertaken in the framework of the Interreg Central Europe project ‘foodCIRCUS’, investigates food preparation, consumption, and waste generation in six educational institutions (three primary schools and three kindergartens) in Wrocław, Poland. Monitoring was done during five consecutive days in each institution (October–December 2024), covering food produced and eaten as well as unserved food, serving leftovers, and plate waste for the main lunch components (soup, protein, starch, and vegetables). The main results give an overall amount of food waste of 236 g/meal planned, with a wastage level of 45%. This is higher than values reported in the literature and higher than the commonly cited global estimate of approximately 30% across the entire food supply chain. Most of the wastage occurred as plate waste (27% of the total food prepared), whereas unserved food constitutes 18%. Primary schools show higher levels of unserved food due to variability in meal uptake, whereas kindergartens show higher plate waste. Vegetables and certain starch-based dishes (groats) have the highest wastage levels. It can be concluded that both operational and behavioral factors cause food waste in school canteens. The national nutrition guidelines lead to a consistent overproduction of food in kindergartens and primary schools, both with their own kitchens and served by catering companies. Reduction strategies should focus on behavioral approaches such as nudging or awareness-raising and operational optimization (e.g., improved forecasting and portion control), as well as redistribution approaches for surplus. The national nutrition guidelines should be interpreted and implemented less strictly. Each pupil should be given the chance to eat portions according to the amounts based on the guidelines, but this does not mean that these amounts have to be prepared for all the pupils potentially attending lunch (as for many it is not their only nutrition source).

## 1. Introduction

Food waste has been identified as a major global challenge, with significant environmental, economic, and social implications. It is estimated that approximately one-third of all food produced for human consumption is lost or wasted at some point in the food supply chain, corresponding to around 30% at a global level [1]. While losses occur at all stages—from production to consumption—the last stage, particularly in institutional settings, has been identified as a critical point for intervention [2].

School lunches are an important part of institutional food services, as they provide meals to a large part of the total population on about 200 days a year. Previous studies have shown that food waste levels in school canteens are substantial, varying from 20% to 45% of food produced [3,4,5,6,7,8,9]. The level of food waste is influenced by both the way the meal providers work and how the pupils behave during school lunches.

In Poland, school meals are regulated by national nutrition guidelines that define daily nutrition intake, meal composition, and how often specific food groups, like vegetables, whole grains, and protein sources, should appear on the menu [10,11,12,13]. However, school pupils’ preferences are often different from what’s recommended, as they tend to avoid the healthier options such as vegetables and wholegrain products. In addition, the guidelines suggest what share of the daily intake should be covered by children’s lunch. On the school menus, the guideline’s calorie-based amounts are transformed into mass portions, which are consequently controlled by the sanitation inspection. However, no alternative sources of nutrition (lunches at home after returning from school, various forms of snacks, etc.) are being taken into consideration. As a result, discrepancies between prescribed menus and consumption behavior may contribute to increased waste generation in the school canteens.

Food waste in schools typically consists of two main factors: unserved food (kitchen surplus) and plate waste. The relative importance of these factors depends on organizational factors (such as attendance variability and serving systems: catering or own kitchen, freedom of choice) and behavioral factors (less liked meal components remain on the plates) [6,7,14,15,16]. Similar patterns have been described in studies on other-than-school catering and food service systems [2,17].

At the same time, there is little national data on how planning practices, serving systems, nutritional guidelines, and the pupils’ preferences lead to varying levels of food wastage. The relation between school type, kindergarten or primary school, and food waste amounts, including the division between unserved food and plate waste, is merely described in the literature. For effective food waste reduction strategies, such as behavioral interventions and operational improvements, more detailed, context-specific analyses of food wastage are needed [18,19,20,21,22].

The present study addresses these gaps by analyzing food preparation, consumption, and waste generation in six educational institutions in Wrocław, Poland, including three primary schools and three kindergartens. The study focuses on (i) the quantification of total food waste and its distribution between unserved food and plate waste, (ii) differences between institution types, and (iii) the role of individual meal components and dishes in the total waste amounts.

By giving component-specific data on food production and wastage in educational institutions, this study contributes to a better understanding of the mechanisms driving food wastage in schools and provides insights for developing targeted measures for its abatement.

## 2. Materials and Methods

This study was undertaken within the framework of the foodCIRCUS project (circular solutions for keeping food waste out of Central Europe’s schools) under the EU Central Europe Program.

### 2.1. Study Design and Preparation

Apart from Wrocław University of Environmental and Life Sciences (UPWr), the Wrocław City Council is also a partner in the foodCIRCUS project. The selection of educational institutions was done by the City Council, based on an estimation of the school’s willingness to participate as well as requirements of representativeness. Both institutions are primary schools and kindergartens, with caterers or their own kitchens.

Initial meetings were conducted in September and October 2024 with representatives of the participating schools and the Polish project partners. The purpose of these meetings was to present the study design and data collection procedures, and to obtain institution-specific information required for the planning of the monitoring activities and subsequent interventions (the latter are not part of this study).

During the monitoring phase, no active involvement of school or catering staff in data collection was required (it was assumed that the staff would simply not have the time available for doing so). However, kitchen personnel facilitated access to production processes and supported the measurement of food quantities where necessary.

### 2.2. Data Collection

Data on food waste were collected between October and December 2024 in six educational institutions in Wrocław, Poland: three primary schools (P1, P2, P5) and three kindergartens (K3, K4, K6). Each institution was monitored over five consecutive school days, excluding weeks with holidays or non-teaching days. Polish schools prepare their lunch menu under the dietary restrictions of the sanitation inspection. Amounts of ingredients and food components are supposed to meet the regulations based on weekly average values (e.g., vegetables, fish, proteins, fat, etc.). Accordingly, a five-day monitoring period was considered appropriate, as it captures a complete school-week menu cycle and aligns the food waste assessment with the weekly framework used to evaluate compliance with nutritional and food-group requirements.

Data collection was carried out by members of the Wrocław University of Environmental and Life Sciences (UPWr) project team, supported by trained student assistants. By not involving school and catering staff in the measurements, researcher bias was prevented. Excluding school and catering staff from the measurements prevented researcher bias. In primary schools, data were collected at a school-wide level, as meals were consumed in a central canteen across mixed classes and time slots. In kindergartens, however, data were collected at a class level, as pupils eat their lunch in their classrooms. Both food produced and leftovers were monitored: before entering (production) and after leaving the classrooms (plate waste and serving waste).

### 2.3. Measurement Approach

Food flows were monitored across three stages of the catering process:

- Food preparation (production).
- Unserved food (distinguishing between kitchen surplus and serving leftovers).
- Plate waste.

For each stage, data were recorded separately for the following components:

- Soup.
- Second dish (protein component: meat or fish).
- Second dish (starch component).
- Second dish (vegetable component).

Desserts and beverages were also observed but excluded from further analysis due to their limited relevance for total food waste and its environmental effects (e.g., drinking water) and difficulties in quantification (e.g., fruits consumed outside the canteen).

In primary schools, the leftover food on the plates returned by pupils was divided by the project staff into the considered meal components. In the kindergartens, only the “food ladies” had access to the classrooms; they collected the leftovers according to meal components in small containers. The mass of prepared food and food waste at each stage and for each component was determined using calibrated kitchen scales. All measurements were recorded as net weights, excluding container mass.

### 2.4. Data Processing

Data were recorded manually using standardized recording sheets. Although the use of a digital monitoring tool was initially considered, it was not implemented. This decision was based on two factors: (i) manual data collection had already started prior to tool acquisition, and (ii) site-specific differences in operational procedures required flexible adaptation of the data collection approach. Such flexibility could not be ensured using a predefined commercial monitoring system.

Based on the weighted total daily amounts of prepared food, food not served, and plate leftovers for all schools, the weekly mean values were determined by dividing the total amounts by the number of meals planned (i) and the number of pupils actually attending lunch (ii). The amounts of food eaten were determined by the difference between the total amounts of food prepared and wasted (food not served and plate leftovers combined). To summarize the wastage levels for all schools, the arithmetic average was used.

For the calculated mean values of food wastage levels, the standard deviation is provided (mean ± SD), together with the data range: minimum and maximum values observed in the considered educational institutions. Differences in food wastage levels between primary schools and kindergartens as well as between food preparation methods were assessed applying a Student’s *t*-test. Differences in to the overall wastage levels of the various food components were assessed applying a one-way ANOVA test. Statistical analyses were performed using Microsoft Excel.

### 2.5. Educational Institutions

A total of six educational institutions were included in the study, comprising three primary schools and three kindergartens (Table 1). Among these, one primary school and two kindergartens operated in-house kitchens, where meals were prepared by school-employed staff. The remaining institutions were supplied by external catering providers.

In most cases, catering companies utilized the existing school kitchen facilities to portion and serve the meals. In some cases, part or all of the meals were prepared on site. Only specific diet meals (e.g., gluten-free or dairy-free) were delivered as pre-packaged individual portions. In the in-house kitchens, the specific diet meals were prepared separately by the kitchen staff.

Meal distribution practices differed between institution types. In primary schools, meals were served to pupils via a central serving counter (“window”) in the canteen. In kindergartens, however, meals were distributed to individual classrooms, where children consumed the food.

An overview of the participating institutions is provided in Table 1.

**Figure 1:**
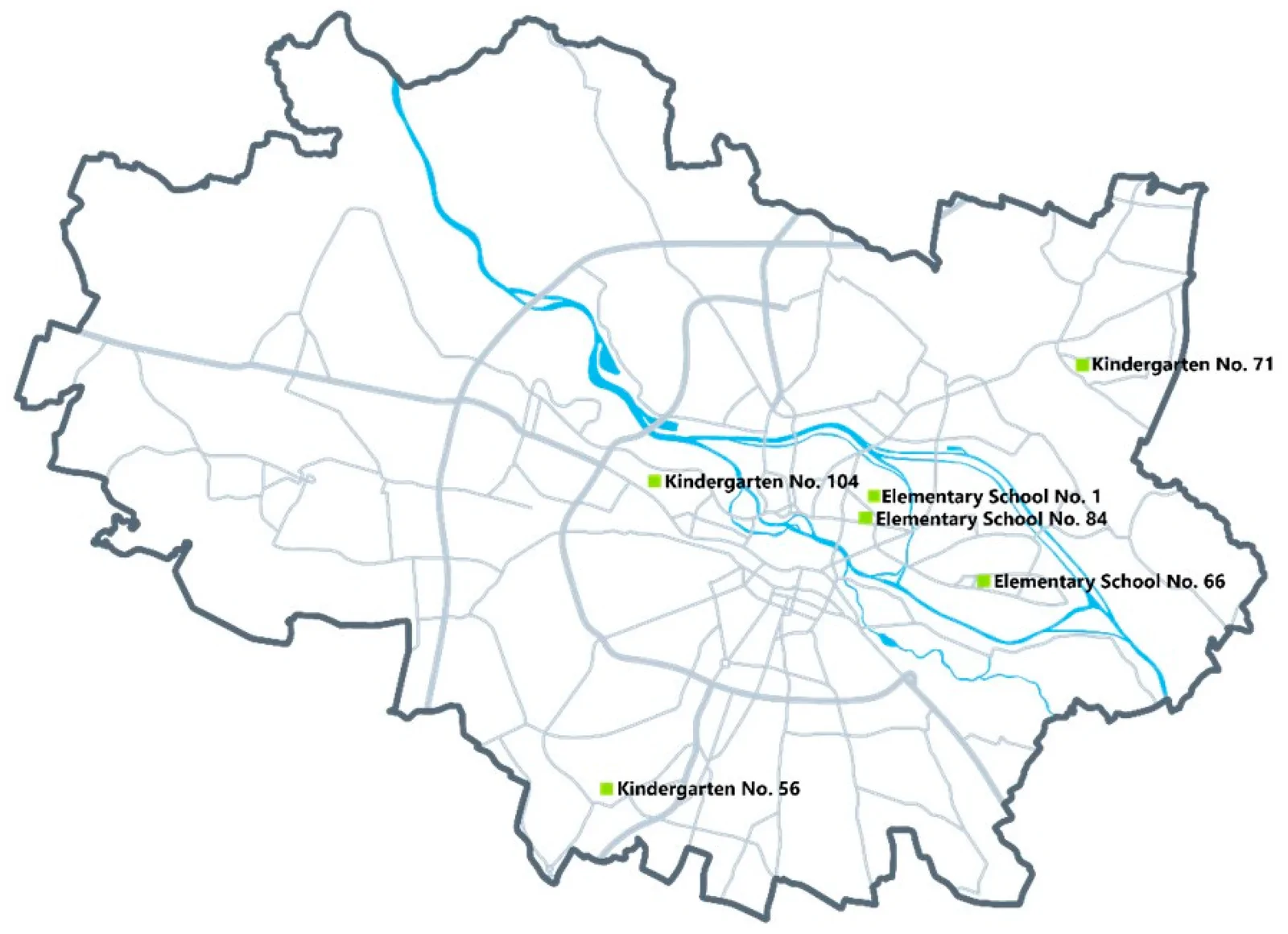
Geographical localization of the schools selected in the city of Wrocław, Poland.

Figure 1 shows the locations of the considered educational institutions in the city of Wrocław. Typical city center and outskirts schools were both considered. A location closer to the city center is practical for later reduction measures in the form of the redistribution of possible surplus food in the schools, as charitable organizations (which would accept and distribute the surplus food) are mainly located in the vicinity of the city center.

## 3. Results

Table 2 gives an overview of the number of pupils in the schools considered and their participation and absence levels.

In primary schools, pupils in older classes attend lunch individually, not accompanied by a teacher. Younger children and kindergarten pupils eat their lunches under the supervision of their teacher.

Absence levels, defined as the share of pupils within the school lunch program not present at lunch on a given day, were generally low in kindergartens (0%). Wrocław kindergartens are equipped with an electronic pupil registration system, which registers how many pupils are attending kindergarten on a given day. After breakfast, this number is corrected for children leaving early or coming late, which gives a precise basis for lunch planning. On the other hand, in primary schools, no such system is in place, and pupils, especially in the higher classes, have the possibility to skip lunch, as they attend lunch independently. Consequently, higher absence levels were observed in primary schools, particularly in P2 (9%) and P5 (25%), suggesting that this may be a contributing factor to overproduction of food.

### 3.1. Food Prepared and Eaten

**Figure 2:**
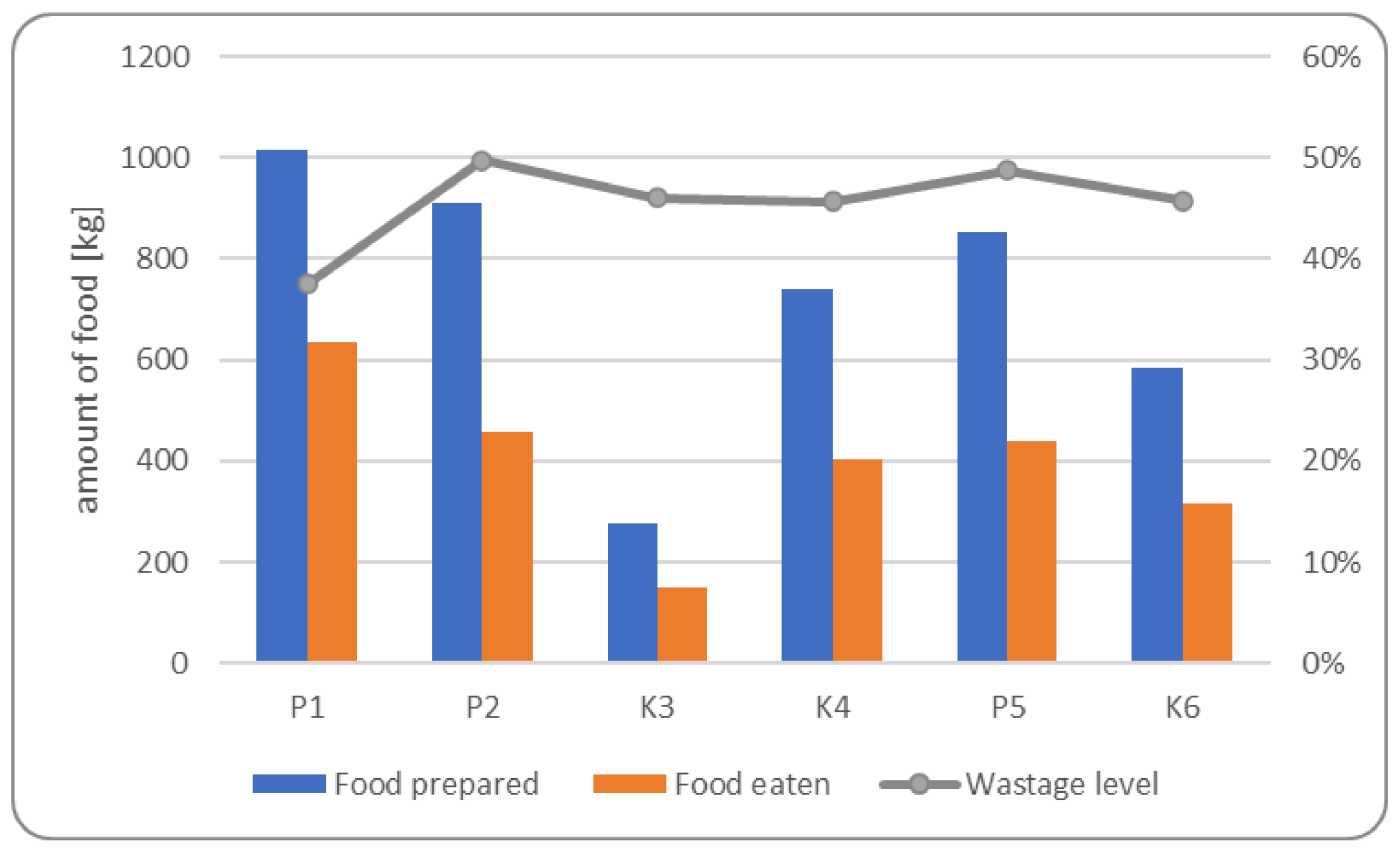
Food prepared and food eaten, as well as wastage level in six educational institutions in Wrocław during a five-day monitoring campaign.

In Figure 2, the overall amount of food produced and eaten is shown, as well as the average wastage level. The data is based on a 5-day monitoring period in three primary schools and three kindergartens.

In all institutions, more food was prepared than was eaten, thus leading to the occurrence of food waste. In primary schools (P1, P2, P5), more food was produced than in the kindergartens (K3, K4, K6), but at the same time, the absolute consumption of food was also higher. The main reason for this lies in the higher number of pupils attending the school lunches in primary schools, whereas kindergartens are generally smaller.

Food waste levels, defined as the overall share of produced food that is not eaten, varied between approximately 38% and 50%. Primary school P1 exhibited a substantially lower wastage level (38%) compared to the other institutions, which ranged from 45% to 50%. This anomaly was attributed to irregularities in the kitchen management, specifically regarding the volumes of soup produced and consumed. In the following section, more details on this matter are provided. Kindergartens (K3, K4, K6) showed very similar wastage levels, slightly lower than in primary schools P2 and P5. Overall, the results indicate that a substantial proportion of the prepared food is not consumed.

### 3.2. Plate Waste and Unserved Meal Components

**Figure 3:**
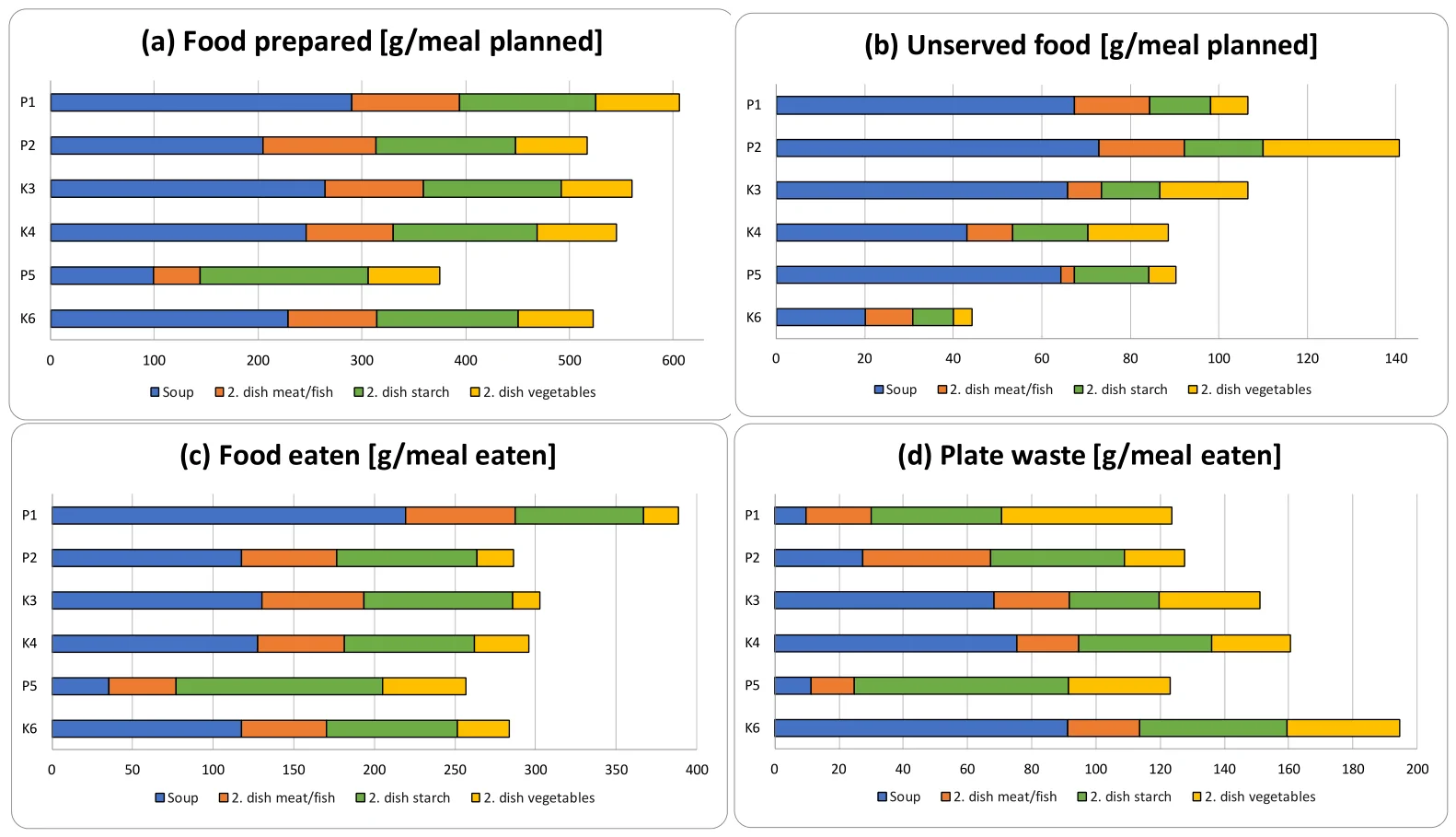
Food prepared, wasted, and eaten in six educational institutions in Wrocław during a five-day monitoring campaign.

In Figure 3, the food wastage situation is presented from the planning/kitchen perspective (a) and (b), with the amounts based on the planned meals, and from the eating/pupils perspective (c) and (d), with the amounts based on the meals eaten.

Generally, mostly soup and starch-based side dishes were prepared in all schools (Figure 3a). Primary schools showed higher total quantities than kindergartens. The prepared amount of the protein-based second dish (meat/fish) was similar in all institutions, with only P5 showing a significantly lower amount. In this primary school, during the week of monitoring, there was a problem with the school’s meat supplier, resulting in more meatless dishes.

Figure 3b shows the unserved food (including serving leftovers), which varies between both institutions and food categories. The overall highest levels were observed in P2, mainly for starch dishes and soup. On the other hand, kindergartens (K3, K4, K6) generally showed lower amounts of unserved food, especially for soup. This is for two reasons. Firstly, kindergartens have a pupil registration system in place; the number of present pupils is known (and even corrected based on the breakfast attendance numbers). All pupils take part in lunch. In primary schools, there is a discrepancy between the planned number of meals and the actual number of pupils eating lunch, which explains the higher amount of unserved food. Secondly, in primary schools, soup is optional, especially for the older pupils, leading to higher amounts of leftover (unserved) soup, as many pupils only take the second dish.

The amount of food eaten (based on the pupils actually attending lunch) per meal (Figure 3c) was the highest in P1. Especially the consumption of soup was much higher compared to the other institutions. Suspicion of irregularities was later confirmed by the school director. It appears that soup was sold to school staff in jars, bypassing the monitoring team. In general, kindergartens showed a tighter range of consumption levels (app. 250–300 g/meal) than primary schools (both the highest and lowest intake were in primary schools).

Figure 3d shows considerable variability in the plate waste amounts. Kindergartens showed higher overall plate waste levels than primary schools, whereas the level of unserved food was lower. This is caused by the serving practice in kindergartens: all or most of the prepared food is divided onto carts for the individual classes. Inside the classes, depending on the individual approach of the serving ladies, most of the food is divided onto the plates. Some food returns as unserved; most is left over on the plates. Vegetables and starch were the main causes of plate waste in most schools. School P5 showed higher amounts of starch both eaten and as plate waste. High amounts were put on the plates, of which a considerable part was consequently not eaten. This is due to the circumstances that, in the monitoring period, there was a problem with the school’s meat delivery; the menu was largely meatless, with higher amounts of starch and meat being replaced by other protein sources. Soup plate waste is lowest for all primary schools, since the soup is provided as an option; many pupils do not take it. Therefore, only those pupils feeling like soup return plates, which are on average emptier than in kindergartens, where all pupils get served soup, and the uneaten soup shows up as plate waste.

Overall, the results show that uneaten food occurs both as unserved food and as plate waste, although their relative shares differ among the institutions studied. Kindergartens showed a lower proportion of unserved food than primary schools, but a relatively higher proportion of plate waste. Primary schools, in turn, were characterized by higher levels of both food preparation and consumption. Concerning the food production system, no clear pattern in food waste levels was observed across the institutions. Despite differences in how meals were prepared and provided, the overall level of food waste was relatively consistent, ranging from 45% to 50%. Therefore, the present results do not provide sufficient evidence to conclude the influence of the food production system on food waste levels.

In Table 3, the average total amount of wasted food in all schools is shown, as well as the relative wastage level.

The average total amount of food waste for all institutions was 236 ± 28 g/meal planned, which means an overall wastage level of 45%. The standard deviation of 28 g/meal planned (app. 12% of the mean value) indicates that the total amount of wasted food is consistent amongst all six educational institutions. Most of the wastage occurred as plate waste (27% of the total food prepared), whereas 18% of the prepared food was kitchen surplus (unserved food and service waste).

When considering the absolute total amount of food wasted in the individual educational institutions, it turns out there is no significant difference between primary schools (average value of 222 g/meal planned) and kindergartens (average value of 248 g/meal planned). Although the wastage level was approx. 12% higher in kindergartens, this difference was not statistically significant when applying Student’s *t*-test (*p* = 0.300).

Considering the preparation method, own kitchen versus catering (the type of catering differs; in some institutions (part of) the food is prepared in the school’s kitchen by the catering staff), a similar result for the absolute total amount of wasted food was achieved. Educational institutions with their own kitchens show an average wastage level of 223 g/meal planned, whereas the involvement of catering companies leads to a wastage level of 247 g/meal planned. Although the wastage level was approx. 11% higher for the food prepared by caterers, this difference was not statistically significant when applying Student’s *t*-test (*p* = 0.360).

The second dish showed the largest absolute amount of waste (133 ± 18 g/meal planned), with a total wastage level of 45%. Within the second dish, starch was the most wasted fraction (55 ± 8 g/meal planned), followed by vegetables (45 ± 10 g/meal planned) and the meat/fish component being the smallest (33 ± 14 g/meal planned). The latter meal component shows a relatively large spread (13–55 g/meal planned), with a standard deviation of 14 g/meal planned (41% of the mean value). This indicates considerable differences in the consumption level of meat/fish amongst the schools. Over a hundred grams of the prepared soup was wasted (102 ± 24 g/meal planned). This constitutes a wastage level of 46%, which is comparable to the level of the second dish wasted.

The soup is wasted mainly as unserved food, never reaching the pupils’ plates (25% of food prepared). The level of soup plate waste is lower at 21%. For the second dish, plate waste is the dominant form of wastage. For vegetables, total plate waste is the highest (42% of food prepared), leading to an overall waste rate for vegetables of 62%. For the protein (meat/fish) component, the total wastage level is the lowest, at 39% of the food prepared. Here, the plate is 25%, whereas the unserved food accounts for 13% of the prepared food amount.

Applying a one-way ANOVA to the overall wastage levels of the various food components shows that the differences between the components are significant, with a *p*-value of 0.0021, which is well below the threshold of 0.05. The F-ratio of 6.995 suggests that the variance between the food components’ wastage levels is nearly 7 times larger than the variance within the food components themselves. However, considering the individual food components, it can be stated that the differences in wastage levels were as follows:

- The difference between the vegetable dish and the meat/fish dish is highly significant (*p* < 0.01);
- The difference between the vegetable dish and the starch dish is significant (*p* < 0.05);
- The difference between soup and other components is not statistically significant (because of the high variation in the soup wastage levels and the low number of observations).

In general, it can be stated that most of the food that is wasted can be found on the pupils’ plates; this is especially true for the vegetables and starch-based components. For soup, more food remains unserved than is left over on plates.

### 3.3. Preferences for Individual Dishes

In the following figures, the shares of individual dishes that were eaten are presented. These are based on the menu served in the schools on each of the days the monitoring lasted. For each dish, the corresponding school number is given.

**Figure 4:**
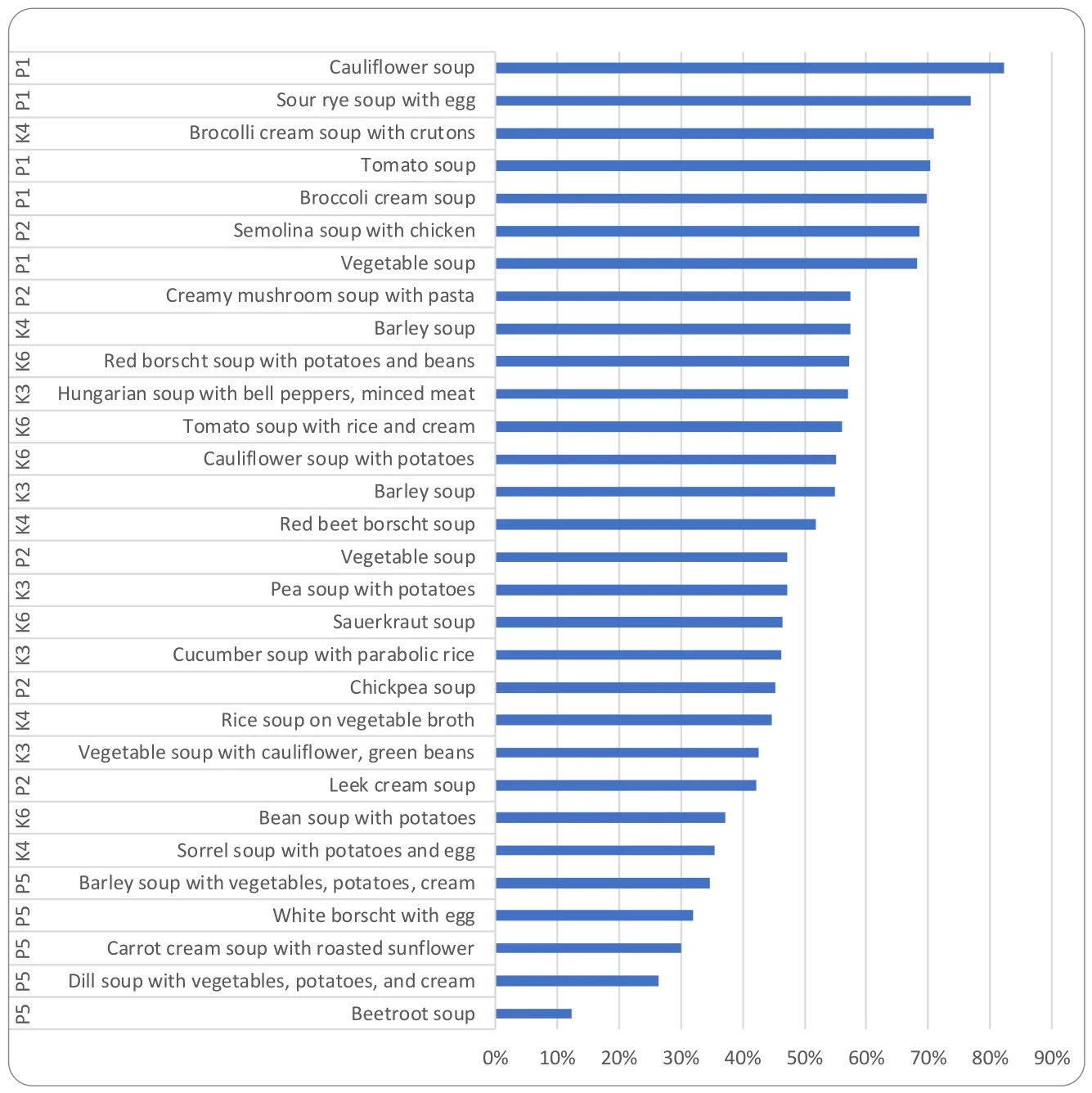
Share of soup eaten in the six educational institutions.

As shown in Table 3, the average waste level for soup is similar to that of the second dish. Figure 4 shows considerable variation in the share of soup eaten, with values ranging from approximately 12% to over 80%. The highest consumption rates were observed for cauliflower soup and sour rye soup in P1, followed by broccoli cream soup with croutons in K4. In general, soups served in P1 were among those with the highest shares eaten, with all observations for this school exceeding approximately 70%. For most soups served in kindergartens and in P2, the share eaten ranged from approximately 40% to 70%, although substantial variation was observed across individual soups. No clear pattern related solely to the type of soup could be identified. In contrast, all soups monitored in P5 were among those with the lowest consumption rates, with only approximately 12–35% of the prepared amount being eaten. These findings may have been caused by the serving practices. In kindergartens, all children get served a portion of soup, whereas in primary schools, the pupils are freer in their decision. In P2 and P1, the teachers often pour the soup for the younger classes; in P5, all pupils have to actively take the soup themselves. This is reflected in the soup consumption rate in P5, which shows the lowest values (ranging from 12% to 35%).

**Figure 5:**
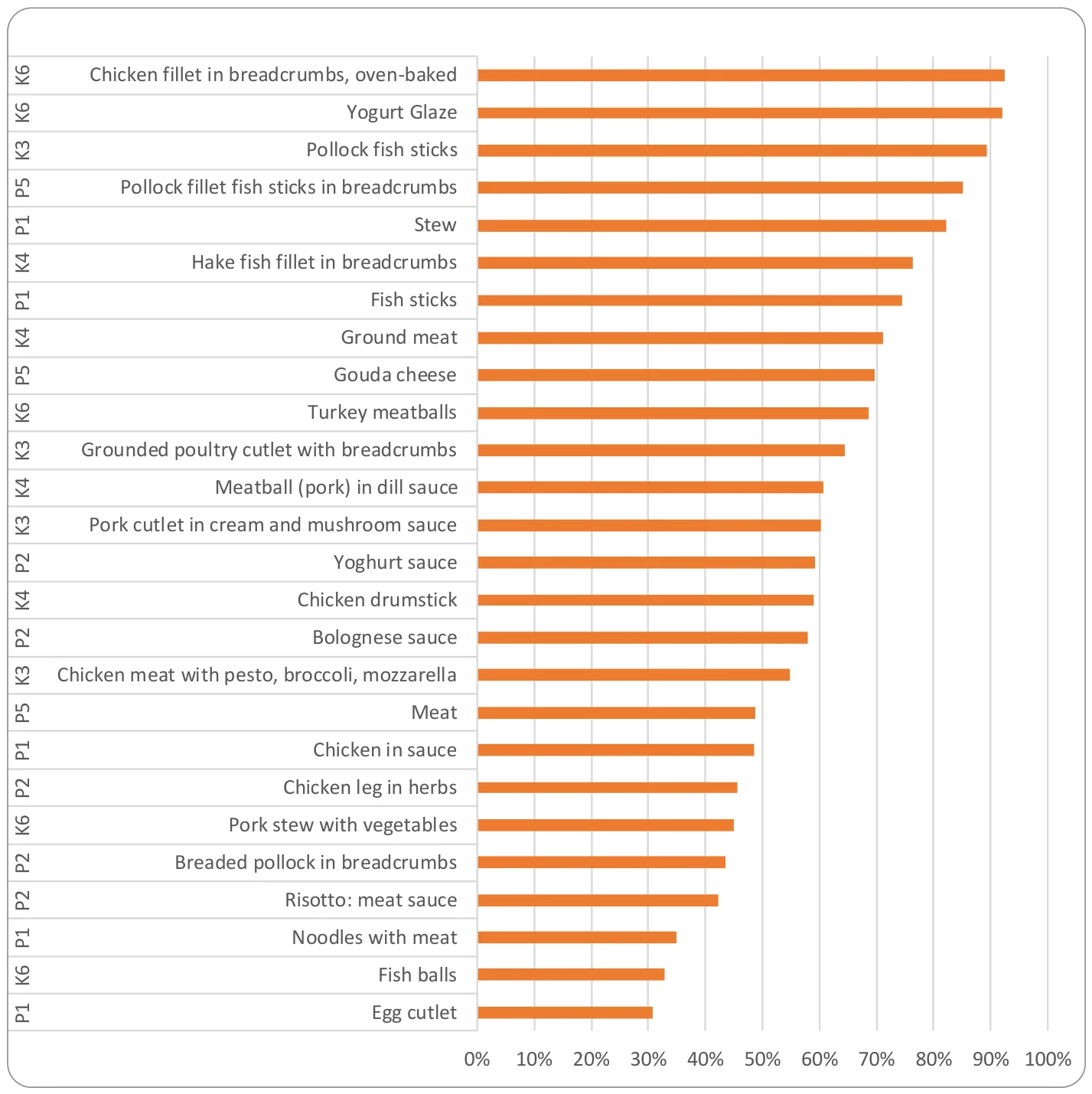
Share of meat/fish dishes eaten in the six educational institutions.

Figure 5 shows the consumption level of the meat and fish components of the second dishes. This component showed the lowest overall wastage level, which is also reflected by the fact that there are eight dishes with a share of dishes eaten of over 70%. Half of these were fish dishes. Meat sauces of a consistency that is difficult to recognize and not-everyday ball- and burger-shaped dishes had the lowest levels of edibility. As for the soups, the acceptance of the meat and fish dishes by the pupils seemed to depend not only on the type of soup but also on how it was prepared. Fish, especially, was eaten much better when it was breaded. Compared to the other dish components, the meat/fish part was, in general, the best eaten. None of the individual schools stand out positively or negatively.

**Figure 6:**
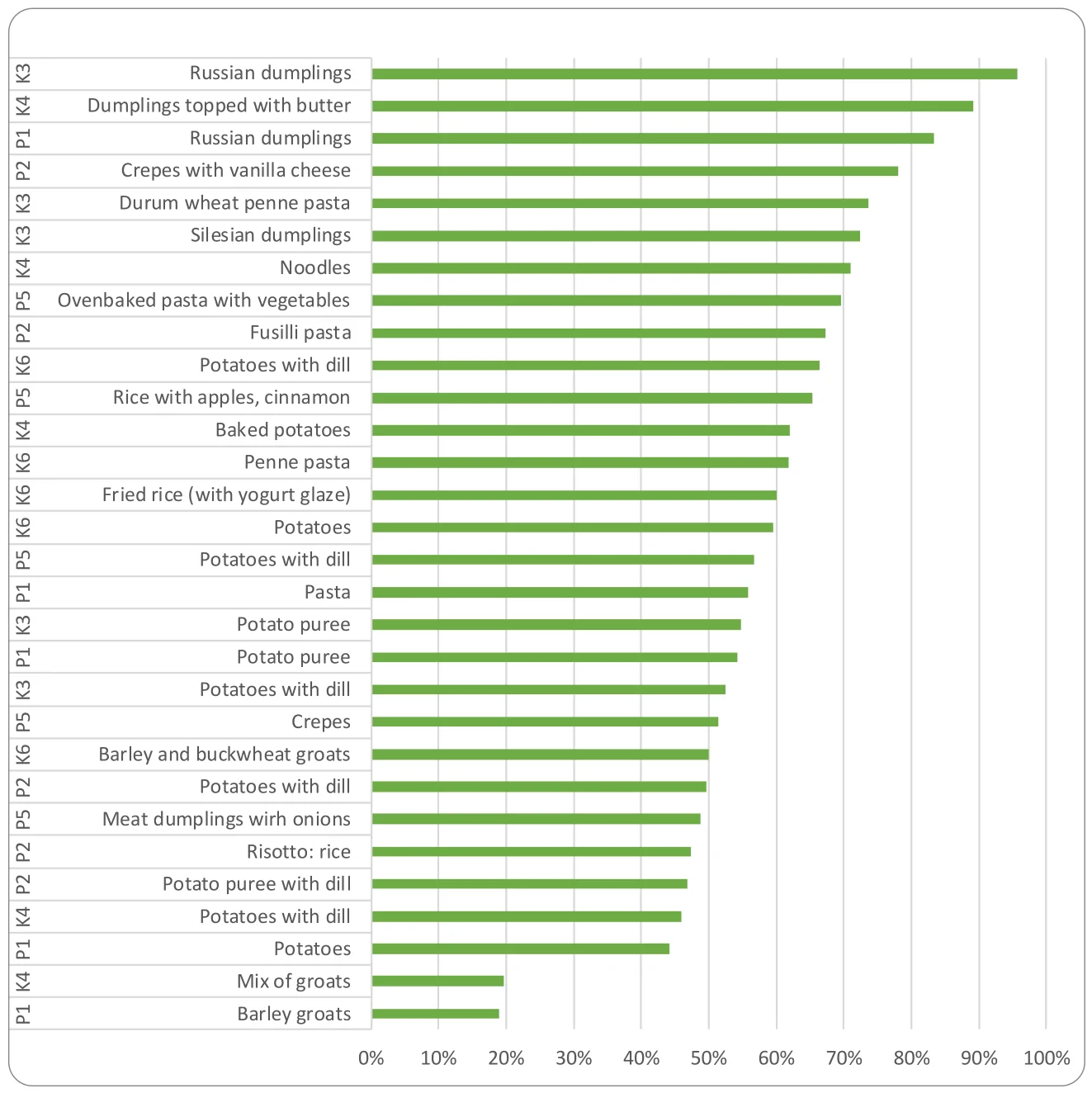
Share of starch dishes eaten in the six educational institutions.

Starch-based side dishes, shown in Figure 6, like the protein-based ones, were generally better eaten than the other components. Groat-based dishes showed little acceptance by the pupils, with consumption levels below 20%. All other dishes had consumption levels exceeding 45%. Potato dishes showed up in various forms, generally with a low to average level of eating rate. Dumplings, on the other hand, were generally liked, with only the meat variant somewhat less popular. In the case of starch dishes, no specific kitchen or caterer stands out.

**Figure 7:**
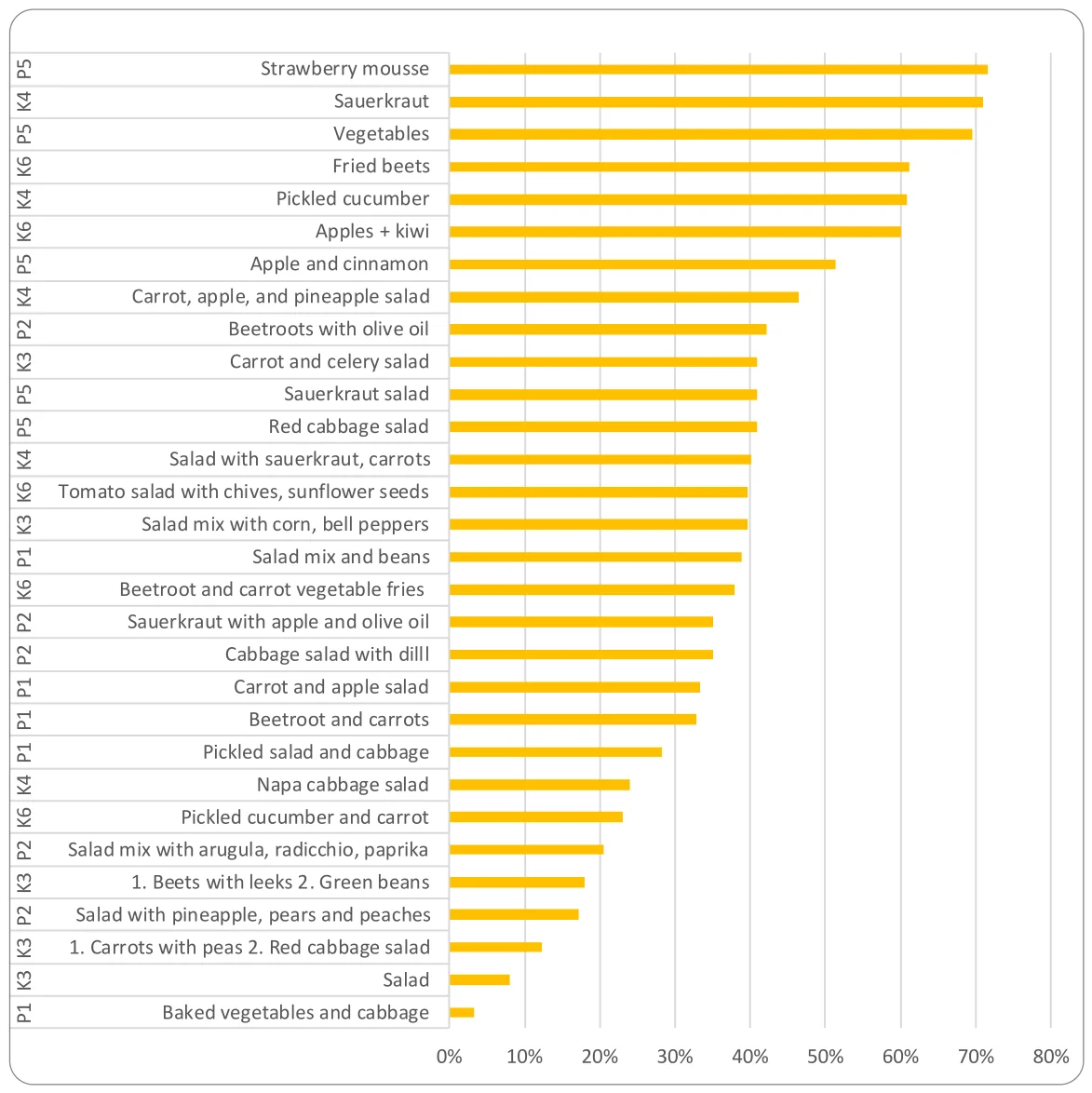
Share of vegetables eaten in the six educational institutions.

The vegetable dishes shown in Figure 7 have the lowest consumption rate of all. Fruits are more widely accepted than most vegetables, with three out of seven dishes above 50% acceptance consisting of fruit. Both the worst and best eaten dishes, as well as several average ones, constituted cabbages in different varieties. Amongst the worst-performing dishes were fried, baked, and cooked vegetables, whereas salads seemed to do better. Overall, about two-thirds of the vegetable dishes had an eating rate close to or clearly below 40%. This was also reflected in the overall numbers in Table 3, which shows the highest plate waste amounts for vegetable dishes. Considering specific institutions, it has been proven that K4, K6, and P5 show higher consumption levels. In K4 especially, the focus is on the shape and form of the prepared vegetables, drawing on the staff’s experience. Raw and cooked vegetables in the form of sticks led to better consumption rates. On the other hand, schools P1 and K3 show worse edibility results. Both institutions offered two types of vegetables on a served plate, which could not be negotiated over by the pupils, often one raw and one cooked.

Rather than encouraging higher vegetable consumption, this strategy appears to result in the rejection of at least one option, thereby reducing total intake.

Generally speaking, the consumption rate is the highest for soup and the lowest for vegetable dishes. All dishes show a rather large variation in edibility, with the meat/fish dishes being a bit more uniformly distributed. Although some specific dishes are clearly accepted better or worse, many of the main ingredients show a large variety of being eaten well or not. Acceptance of the dishes by the pupils seems to depend not only on the type of dish but also on how it was prepared.

## 4. Discussion

Across all educational institutions, the average food wastage ranges from 45% to 50%, except for primary school P1, which shows a substantially lower wastage level of 38%. The lower level of wastage in P1 was caused by irregularities, especially in soup overproduction and alternative ways of distribution. School P5 also had a non-standard week, as there were problems with the meat delivery. On average, 236 g of the food prepared for each planned meal remains uneaten and is discarded as waste. As a sensitivity analysis, not including the results of P1 and P5, the average amount of food wasted constitutes 250 g/meal planned. Because the impact of excluding P1 and P5 is small and because irregularities are part of daily practice in school catering environments (some of which are not noted by researchers), the following results of all educational institutions are included.

These results place the studied institutions at the upper end of the range reported in previous research on school catering and institutional food services. Earlier studies have typically documented total food waste levels of 100–200 g per meal, corresponding to approximately 20–45% of the food prepared [3,4,5,8,9]. For example, Eriksson et al. reported food waste levels of approximately 120–150 g per meal in Swedish public catering, while Silvennoinen et al. found comparable values in Finnish catering services [4,17]. Studies focusing specifically on school lunches have generally reported lower relative wastage levels, ranging from approximately 25% to 35% of the food served [6,7]. Overall, the institutions examined in the present study exhibited higher levels of food waste than previously reported school catering systems, both in absolute terms (g/meal) and as a proportion of food prepared (%). The only exception is primary school P1, which contrasts with findings from a recent Hungarian study, which reported comparatively high levels of food waste in school meals provided by catering companies [18]. There is very little data available about wastage levels in Polish schools. In a recent study, qualitative measurements were made in a participatory approach (measurement by pupils) in a number of Warsaw schools [23]. The measurements were based on visual observations of plates: empty, one-quarter full, half empty, three-quarters full, or full plates, which were used for the assessment of waste reduction interventions. However, no quantitative (g/meal) or relative (%) wastage levels were determined; also, the production phase was not monitored.

In light of these findings, the magnitude of food waste observed during school lunches is particularly noteworthy. Gustavsson et al. estimated that approximately 30% of all food produced is lost or wasted across the entire food supply chain [1]. By comparison, the present study found that the final stage of the supply chain—food consumption in educational institutions alone—accounts for 45–50% wastage in most of the facilities investigated. This indicates that food consumption during school lunches is associated with exceptionally high levels of waste, influencing the average losses estimated for the entire food supply chain.

In the present study, plate waste accounted for approximately 27% of the food prepared, while unserved food represented 18%. Thus, plate waste constituted the larger share of total food waste, consistent with previous studies reporting that plate waste typically accounts for approximately 50–70% of the total food waste generated in school canteens [6,7]. However, the relative contribution of these two waste factors differed between the investigated institutions. In primary schools, the proportion of unserved food was comparatively high, whereas in kindergartens it was considerably lower due to the consistently high attendance of children during lunch. As a result, plate waste represented a larger share of total waste in kindergartens. In primary schools, many pupils choose not to eat soup, which reduces the amount of plate waste for this meal component while increasing the proportion of unserved food. Similar shifts in the balance between plate waste and kitchen surplus have been reported elsewhere and are largely attributed to the accuracy of meal planning, attendance forecasting, portioning practices, and food acceptance by consumers [2,24]. In the present study, discrepancies between the number of planned and actual meals, together with the practice of serving food from bulk containers rather than as individually portioned meals, appear to be the main factors contributing to the observed distribution of waste. Previous research has shown that in self-service buffet systems, where meals are served by canteen staff from buffet counters rather than being individually pre-portioned, the largest share of food waste originates before consumption as a result of preparing more food than is ultimately served [4]. This serving waste is particularly relevant in Polish primary schools, where school lunches are commonly provided using a self-service buffet model. In such systems, the amount of waste largely depends on how accurately kitchen staff can anticipate pupils’ attendance, portion requirements, and actual food intake. Drawing on interviews with school catering managers, Silvennoinen et al. suggested that these losses could be reduced through more accurate forecasting of meal demand, careful menu planning, and preparing food in smaller batches according to real-time demand [4]. However, the authors also noted that implementing batch cooking may be challenging in practice because it requires sufficient staffing levels, as well as a high degree of commitment and flexibility from food service personnel. Additionally, in Poland, school meals are regulated by national nutrition guidelines that indirectly prescribe amounts of food to be prepared for pupils [10,11,12]. Children often have other nutrition sources aside from school lunches, which is one of the main contributors to the generation of unserved food.

When individual meal components are taken into account, vegetables emerge as the most problematic category, with wastage levels reaching up to 60%. This finding is consistent with previous studies, which also identified vegetables as the meal component generating the highest levels of plate waste [6,7,25]. For example, studies from the United States reported that approximately 30% of vegetables and up to 40% of fruit served in school meals remained uneaten [26,27]. Similarly, Blondin et al., Silvennoinen et al., and Smith and Cunningham-Sabo identified vegetables, fruit, and salads as the meal components most frequently discarded by pupils [4,28,29]. This is particularly concerning given the important nutritional role of vegetables, which are a major source of dietary fiber, vitamins, minerals, and bioactive compounds essential for healthy growth and development [28]. High levels of vegetable waste may therefore reduce the nutritional quality of school meals, especially for children, whose adequate intake of these nutrients is crucial during periods of growth [30]. Interestingly, Ramsay et al. reported an all-or-nothing pattern of fruit and vegetable consumption among kindergarten children, with participants tending to either consume the entire serving or not eat it at all [31]. In this context, a self-service system, i.e., children serve themselves, rather than having meals served by kitchen staff, as in the system described above, has been proposed by several authors as a strategy to reduce vegetable waste and increase vegetable consumption. Allowing children to serve themselves vegetables, choose the portion size, and adjust seasonings according to their preferences may improve the acceptance of vegetable dishes. By giving children greater autonomy over this meal component, self-service can encourage more conscious choices and increase the likelihood that the vegetables selected will actually be consumed, thereby reducing their share in plate waste [31,32].

In contrast to vegetables, soups were associated with relatively low plate waste and higher consumption rates. It should be noted, however, that soups have a high water content and are therefore relatively heavy. Consequently, even small quantities of discarded soup may contribute disproportionately to the total mass of food waste. This aspect is particularly relevant in countries such as Poland and Hungary, where soups and other water-rich dishes (e.g., stews) are commonly served as part of the daily school lunch, potentially increasing the measured mass of food waste despite their relatively high consumption [18].

Differences in the acceptance of starch-based side dishes were also observed. Groat-based dishes showed comparatively low consumption, whereas potato-based dishes performed considerably better, with dumplings being among the most preferred options. These findings are consistent with previous research showing that potato-based dishes are generally associated with higher consumption and lower waste, while alternative carbohydrate sources that are less familiar to pupils and less commonly consumed at home tend to have lower acceptance and higher wastage [14]. The protein component, meat and fish dishes, was generally well accepted in the present study. Notably, most fish dishes exhibited wastage levels below 30%. This agrees with the findings of Verbeke and Vackier, who demonstrated that fish achieve high consumer acceptance when prepared in a familiar and culturally acceptable manner, such as fish fingers [33]. Overall, these results suggest that the acceptance of school meals depends not only on the type of food served but also on how it is prepared and whether it matches children’s habitual eating patterns.

As both the absolute (g/meal) and relative (%) food waste levels observed in the present study were high, the underlying causes are likely to include a combination of operational and behavioral factors, as suggested in previous research [18]. In primary schools, one important operational factor contributing to serving waste is student absences during lunch, which makes it difficult to accurately estimate the number of meals required. Behavioral factors are also likely to play a significant role. Across all investigated institutions, pupils had little or no choice regarding the menu, as meals were predetermined. Although introducing a full menu choice may not always be feasible, even limited opportunities for choice could improve meal acceptance. For example, in the study by Smith and Cunningham-Sabo, pupils were allowed to choose the type of milk served with their lunch. More than 70% of elementary and middle school students selected fat-free chocolate milk, and approximately 60% chose flavored milk [28]. The authors concluded that offering fat-free flavored milk enabled schools to meet nutritional standards while remaining within recommended limits for fat and energy intake, demonstrating that even small elements of choice can encourage the selection and consumption of healthier food options.

Furthermore, particularly in kindergartens and among younger primary school pupils, opportunities to refuse individual meal components or request smaller portions were limited, which may have contributed to higher levels of plate waste. Finally, the acceptance of some menu items appeared to be low. Traditional dishes, such as groats or cooked vegetables, which are less familiar from children’s home diets, were associated with particularly low consumption and consequently higher waste. For these meal components that are less appealing to children, greater attention should be paid to their sensory characteristics during school menu planning. In particular, the appearance, texture, and flavor of these foods should be carefully considered to improve their acceptance. Petchoo et al. further suggested that meals should include an attractive combination of foods presented in different shapes and sizes, such as cubes, strips, or bite-sized pieces [34]. In addition, the visual arrangement of meal components on the serving tray may enhance the attractiveness of the meal, particularly for younger children. The offering of two types of vegetables, a cooked one and a raw one, leads to higher wastage levels overall. A choice between two options would likely work better, as in that case the least preferred option would not be served and consequently would not have to be prepared.

The results also suggest that pupils’ preferences should be taken into account when determining the variety of foods offered. Limiting the provision of options that are consistently poorly accepted may help reduce food waste. Adapting menu planning to observed consumption patterns and preferences, while maintaining nutritional requirements and appropriate dietary variety, may contribute to reducing food waste in school canteens.

Given the high levels of food waste observed, effective reduction measures should be implemented. Potential intervention strategies include behavioral approaches, such as nudging and awareness-raising initiatives, as well as operational measures, including improved demand forecasting, portion size optimization, and the redistribution of surplus food. These approaches have also been widely discussed in the recent food waste literature [19,20,21,35]. Future research should focus on evaluating the effectiveness of these interventions, considering both operational improvements and behavioral measures. Such research would provide a stronger evidence base for developing effective strategies to reduce food waste in school lunch programs. In the framework of the foodCIRCUS program, a redistribution pilot is being implemented in two Wrocław primary schools, where the amounts of not-served food are higher. Furthermore, national guidelines should clarify that while every pupil should have the opportunity to receive portions that comply with nutritional recommendations, this does not imply that these quantities must be prepared for all pupils who may potentially attend lunch. This has been brought to attention nationally during a meeting with the Parliamentary Commission for Combating Food Waste and the National Institute of Public Health.

Several limitations of this study should be acknowledged. First, the participating educational institutions were not selected using a completely random sampling procedure. The schools and kindergartens were selected in cooperation with the Wrocław municipality based, among other factors, on their willingness to participate in the foodCIRCUS project. However, the willingness to participate was only the last requirement that led to the final number of participating schools. The requirements communicated to the municipality specified that the study group should collectively represent all of the following characteristics: kindergartens and primary schools; institutions with both in-house kitchens and catering services; institutions located both in the wider city center and in the outskirts; and institutions situated in both high-rise and low-rise residential areas. Consequently, this sample should be considered “predefined” only in the sense that it is not stratified relative to the entire population of schools; therefore, averaging the results may entail a slight margin of error. The six institutions participating in the study can be regarded as statistically representative of all schools and preschools in Wrocław—though not of Poland as a whole—and the findings should not be generalized to the broader population of educational institutions. Rather, this study should be interpreted as a case study providing detailed empirical evidence regarding six institutions operating within a specific local context.

Second, all participating institutions were located in Wrocław, a large Polish city. Although national regulations and nutritional guidelines concerning meals served in schools and kindergartens apply across Poland, the organization of school catering, available infrastructure, meal preparation and serving practices, socioeconomic conditions, and pupils’ eating habits may differ between large cities, smaller towns, and rural areas. These contextual differences may influence both the amount and composition of food waste. Future studies should therefore include institutions from different geographical and socioeconomic settings and, where possible, apply a stratified sampling approach accounting for factors such as institution type, location, size, and catering system.

Another important limitation is the relatively short monitoring period. Food production, consumption, and waste were monitored for five consecutive school days in each institution. While this approach provided detailed information on different food waste streams and individual meal components during the monitored week, a single five-day period cannot capture longer-term variability related to menu cycles, seasonal changes, fluctuations in pupil attendance, special events, or other temporal factors affecting food consumption and waste. The results should therefore be considered a snapshot of food waste generation during the monitoring period rather than an estimate of typical annual food waste levels in the participating institutions. Monitoring over several non-consecutive weeks, preferably covering different menu cycles and seasons, would provide a more robust basis for identifying recurring patterns and distinguishing systematic effects from short-term variation. Nevertheless, a five-day monitoring period is not uncommon in the literature [18,26,28,30,34,36]. Moreover, the preparation of food in Polish schools is based on weekly cycles, in which individual dishes may vary, but the overall types (e.g., fish, pasta, rice, raw/cooked vegetables) as well as the nutritional values (calories, fat, sugar and salt contents) are very similar throughout the year.

These limitations restrict the external validity and generalizability of the findings. Nevertheless, the detailed measurement of food produced, consumed, unserved, left over during serving, and left on pupils’ plates across six educational institutions provides insight into where and how food waste occurs within school and kindergarten catering systems. The findings can therefore be used to identify potential areas for further investigation and intervention, but they should be interpreted within the specific context and timeframe of the present study. Future research based on larger, random, or stratified samples and longer, repeated monitoring periods is needed to determine whether the patterns identified here are also observed across other educational settings in Poland.

## 5. Conclusions and Recommendations

The findings of this study indicate that food waste in school canteens is not solely a consequence of pupils’ food preferences but also reflects how meals are planned, portioned and served. From a practical and managerial perspective, reducing food waste therefore requires a more demand-responsive approach that considers actual attendance, observes consumption patterns and pupils’ acceptance of individual dishes, while maintaining nutritional requirements. Importantly, the two main waste streams identified in this study require different management responses: unserved food can primarily be addressed through improved forecasting and production planning, whereas plate waste requires greater flexibility in portion sizes, serving practices and menu design.

In particular, the present study showed the following:

- On average, 236 g of food per planned meal was wasted in the investigated primary schools and kindergartens.
- This corresponds to a food waste level of 45–50% of the planned meal, except for school P1 (38%).
- Both observations are high compared with the literature values and with the commonly cited global estimate of approximately 30% food loss and waste across the entire food supply chain.
- The overall amount of food prepared, consumed, and wasted was comparable between kindergartens and primary schools, although the composition of waste differed: primary schools generated relatively more unserved food, whereas kindergartens generated more plate waste. Overall, plate waste exceeded unserved food (27% vs. 18% of prepared food).
- Soups and most protein-based dishes were generally better accepted than vegetables and some starch-based dishes, particularly groats.
- Offering two types of vegetables (a cooked one and a raw one) leads to overall higher wastage levels. A choice between two options would likely work better.
- The findings indicate that current national nutrition guidelines may unintentionally contribute to food waste by encouraging standardized portion sizes that do not reflect pupils’ actual consumption or alternative food sources outside school, while simultaneously requiring nutritionally desirable but less preferred meal components.

The findings of this study provide preliminary indications that reducing food waste in school canteens may require balancing nutritional objectives with operational flexibility, particularly regarding the following:

- Each pupil should have the opportunity to receive a portion consistent with national nutrition guidelines; however, this does not imply that guideline-sized portions need to be prepared for all pupils attending lunch, as school meals are not the only substantial meal consumed during the day for many children.
- Schools should introduce more flexible portioning systems, allowing pupils to choose portion sizes while remaining compliant with nutritional recommendations; in particular, smaller initial serving sizes, with the possibility of receiving an additional portion, could help reduce plate waste while ensuring that pupils who wish to eat more can do so.
- Menu planning should take into account observed consumption patterns and pupils’ food preferences. Foods that are consistently poorly accepted and generate high levels of waste should be reconsidered, replaced with nutritionally equivalent and better-accepted alternatives, or offered less frequently, provided that nutritional requirements are maintained.
- Where several food options are offered simultaneously, particularly vegetables, providing pupils with a choice rather than automatically serving all options may reduce waste of the least-preferred items.
- Forecasting methods, portion control, and continuous monitoring of plate waste should become part of routine kitchen management to improve production planning.
- Behavioral interventions, including nudging and awareness-raising activities targeting both pupils and staff, should be implemented and evaluated.
- Where regulations permit, redistribution strategies for safe surplus food should complement prevention measures to further reduce avoidable waste.

Future studies should evaluate the effectiveness of these interventions under real school conditions, including behavioral approaches, forecasting tools and flexible serving systems. Longitudinal studies involving a larger and more diverse sample of schools would help assess the long-term impact of operational changes on both food waste reduction and nutritional outcomes.

## Figures and Tables

**Table 1:** Overview of educational institutions involved in the study.

No.	School Name	Number of Pupils	Age	Monitoring Period	Food Preparation
P1	Primary School No. 66	630	7–14	21–25.10.2024	Caterer in the kitchen
P2	Primary School No. 84	797	7–14	04–08.11.2024	Catering
K3	Kindergarten No. 104	250	3–6	18–22.11.2024	Catering + own kitchen
K4	Kindergarten No. 56	380	3–6	25–29.11.2024	Own kitchen
P5	Primary School No. 1	663	7–14	02–06.12.2024	Own kitchen
K6	Kindergarten No. 71	276	3–6	09–13.12.2024	Own kitchen

**Table 2:** Participation of pupils in the school lunches.

No.	Number of Pupils	Meals Planned [av. no./d]	Meals Eaten [av. no./d]	Absence Level [%]
P1	630	336	327	3%
P2	797	353	321	9%
K3	250	98	98	0%
K4	380	272	272	0%
P5	420	455	341	25%
K6	276	224	224	0%

**Table 3:** Average absolute and relative wastage level in the considered educational institutions.

	Total Wasted [g/Meal Planned]			Wastage Level [%]		
Meal Component	Mean	Min.	Max.	Unserved	Plate Waste	Total
Soup	102 (±24)	73	134	25%	21%	**46%**
*2. Dish*	133 (±18)	110	159	14%	31%	**45%**
*2. Meat/fish dish*	*33 (±14)*	*13*	*55*	*13%*	*25%*	***38%***
*2. Starch dish*	*55 (±8)*	*41*	*67*	*10%*	*29%*	***39%***
*2. Vegetable dish*	*45 (±10)*	*30*	*60*	*20%*	*42%*	***62%***
**Total**	**236 (±28)**	183	258	**18%**	**27%**	**45%**

## Data Availability

The original contributions presented in this study are included in the article. Further inquiries can be directed to the corresponding author.
